# Neural Prosthetics: A Review of Empirical vs. Systems Engineering Strategies

**DOI:** 10.1155/2018/1435030

**Published:** 2018-11-07

**Authors:** Gerald E. Loeb

**Affiliations:** Professor of Biomedical Engineering, University of Southern California, 1042 Downey Way (DRB-B11) Los Angeles, CA 90089, USA

## Abstract

Implantable electrical interfaces with the nervous system were first enabled by cardiac pacemaker technology over 50 years ago and have since diverged into almost all of the physiological functions controlled by the nervous system. There have been a few major clinical and commercial successes, many contentious claims, and some outright failures. These tend to be reviewed within each clinical subspecialty, obscuring the many commonalities of neural control, biophysics, interface materials, electronic technologies, and medical device regulation that they share. This review cites a selection of foundational and recent journal articles and reviews for all major applications of neural prosthetic interfaces in clinical use, trials, or development. The hard-won knowledge and experience across all of these fields can now be amalgamated and distilled into more systematic processes for development of clinical products instead of the often empirical (trial and error) approaches to date. These include a frank assessment of a specific clinical problem, the state of its underlying science, the identification of feasible targets, the availability of suitable technologies, and the path to regulatory and reimbursement approval. Increasing commercial interest and investment facilitates this systematic approach, but it also motivates projects and products whose claims are dubious.

## 1. Introduction

Neural prosthetics are the clinical application of the science of neurophysiology and the methodology of electrophysiology. Almost all physiological functions are coordinated and controlled by electrical signals that share strikingly similar biophysical principles and cellular machinery: sensory perception, movement, cognition, emotion, digestion, excretion, endocrine function, blood circulation, cellular immunity, etc. The technology to record and manipulate these electrical signals was used first to identify and understand this physiology and then, often rapidly and even prematurely, to try to mitigate clinical dysfunction. The rush to exploit new scientific knowledge even as it remains uncertain and incomplete is understandable. Much of modern medical practice owes its existence to serendipity that subsequently motivated systematic scientific inquiry rather than the inverted and largely fictional version of the scientific method taught to students. The value of the occasional successes of Edisonian empiricism (https://en.wikipedia.org/wiki/Edisonian_approach) has often outweighed the costs of many failures and tended to undermine the credibility of scientific conservatives. Nevertheless, changing circumstances warrant a reexamination of this frontier mentality.

The accumulated scientific knowledge about most bodily functions has increased hugely, even for subsystems that are not yet the target of neural prosthetic interfaces. Finding this knowledge is both more difficult given its sheer volume and specialization and much easier given powerful search engines and online archives. Both experimental and modeling methods now make it much more feasible to mount a systematic approach to filling in the missing pieces of science and to designing and building prosthetic interfaces that are likely to work. This can be based on rigorous biophysical models of how electrical fields modulate neural activity [1–3] but only if those models have been validated experimentally.

**Figure 1:**
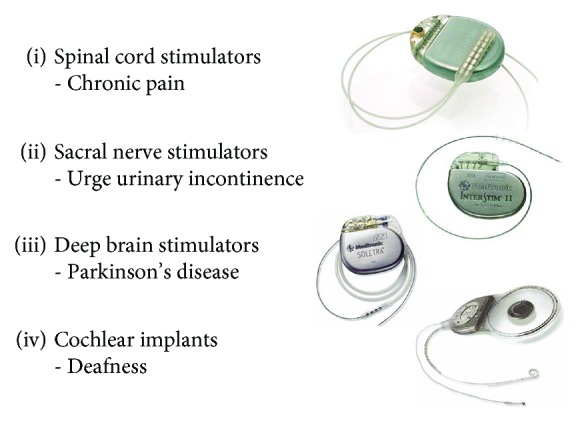
Most currently approved and clinically successful neural prostheses use fairly bulky hermetic titanium cans with multiple feedthroughs and polymerically encased flexible leads to relatively large platinum-alloy electrodes, similar to cardiac pacemakers of the 1970s.

Most of the currently approved and clinically successful neural prostheses described below utilize technology and functionality that is little changed from cardiac pacemakers of the 1970s (Figure 1). Most of the future applications of neural prostheses will require many more densely packed channels of communication into and out of the nervous system. The available armamentarium of technology makes it possible to design and build interfaces that directly address the underlying science, but they also greatly increase the complexity, time, and expense of the product development cycle.

Empiricism is still necessary for demonstrating safety and efficacy of a putative product. Standards for clinical trial design and reporting (e.g., “open data”) are progressing rapidly but are still often ignored, particularly when authors are inventors or advocates for a new technology. The “good old days” of unregulated design, fabrication, and human testing of novel medical devices are gone. Most of the major commercial successes reviewed below obtained their “proof of principle” in those days, whereas all of those still in their infancy must comply with government regulations for Quality Systems (QS), Design Controls, Good Laboratory Practices (GLP), Good Clinical Practices (GCP), Risk Analysis, Investigational Device Exemption (IDE), Pre-Market Approval (PMA), etc.

The near-ubiquity of medical insurance in industrialized countries means that there are few, if any, “early adopter” patients willing or allowed to pay for unproven treatments. This means that even the first versions of new products must immediately meet the high and expensive bar of proving their cost-benefit to third-party payers whose business model mandates high levels of skepticism.

## 2. Case Studies

For the business of neural prosthetics, it is essential to understand the nature of the challenges facing a new undertaking. This means identifying and separating the very different risks that arise from incomplete scientific knowledge, complexity of technology, the economics and politics of healthcare delivery, and the ethics of intervening in behavioral and cognitive functions usually ascribed to free will [4]. The case studies provided below are intended to illuminate the discussion of strategy and tactics.

### 2.1. Pain Control

Transcutaneous electrical nerve stimulators (TENS), spinal cord stimulators (SCS, originally called dorsal column stimulators; Figure 1), and other stimulators for pain control all rely on a mechanism that is familiar to anyone who has ever rubbed the skin around a painful site or used a counter-irritant such as a menthol ointment or mustard plaster to obtain relief. Such phenomena led to the hypothesis and at least partial demonstration of a neural circuit by the Melzack-Wall gate theory of pain in the 1960s [5]. The nociceptors that give rise to painful sensations convey their signals through spinal neurons that project to the brain. These projecting neurons are inhibited by other interneurons that are excited by the low-threshold mechanoreceptive afferents responsible for light touch and vibration sensing in the skin. Electrical stimulation is simply a convenient, selective, and easily controlled means to excite strongly these low-threshold afferents to maximize this inhibition [6]. At the same time, care must be taken to avoid stimulating the equally low-threshold motor nerve fibers that activate muscle contractions and often run nearby. TENS has the advantage of not requiring the surgical implantation required for SCS but the disadvantage of encumbering the patient with skin electrodes and wires and variability in their positioning and efficacy when donned and operated by the patients themselves.

The pain modulating circuits appear to be anatomically quite localized and topographically arranged, giving rise to the various anatomical and technological approaches for directing the stimulation to the desired sensory targets [7]. Such electrical stimulation produces buzzing sensations called paresthesias. These are easily localized by the patient and can be used to guide the placement and selection of electrodes and adjustment of stimulus parameters so that the paresthesias coincide with the perceived location of the pain, thereby maximizing analgesia [8]. When the pain is well-localized to a dermatome, laterally placed electrodes can target specific dorsal root ganglia (DRG stimulation) [9]. Detailed models of the spread of electrical currents through the heterogenous tissues of the spinal column have proven useful in the design and deployment of increasingly complex multicontact electrode arrays and multichannel stimulators [10]. Clinical results are probably approaching the limits whereby steering currents from a distance can selectively activate the many different functional circuits of the spinal cord. Temporal modulation such as burst patterns may provide another way to tailor treatment effects. The empirical findings in patients have motivated basic science that has revealed many neurophysiological nuances, which may afford new therapeutic opportunities [11].

Recently, there has been clinical interest in high-frequency spinal cord stimulation (HF-SCS) that utilizes low-charge stimuli at unphysiologically high repetition rates of 1000–10,000 pulses per second (pps). Stimulation amplitude is selected to produce no detectable paresthesia, so presumably, it is not activating the low-threshold mechanoreceptors that, in turn, activate the pain-inhibiting gate interneurons. Animal studies have yet to provide any plausible physiological mechanism [12]. Clinical studies could take advantage of the lack of sensation to produce blinded control periods or dose-effect stratification but have generally not done so, making it difficult to exclude placebo effects. Such a double-blinded but small clinical study showed no effect over sham-stimulation [13].

Some forms of pain in viscera and pelvis respond poorly to SCS, either because these circuits are lacking in gating interneurons or because their anatomy is not conducive to stimulating the low-threshold afferents that might gate them [14]. If spinal anatomy is the limitation, then it may be possible to target stimulation of peripheral cutaneous nerves [15]. Attempts to treat various forms of headache and facial pain by stimulation of cutaneous afferents such as the trigeminal and posterior occipital nerves have had some success but also problems with delivering electrodes accurately into soft tissues and maintaining them in position despite movement and stress from surrounding neck muscle activity [16–18].

### 2.2. Deep Brain Stimulation (DBS)

Parkinson's disease has long been associated with a lesion in the substantia nigra (pars compacta) of the basal ganglia (BG). Attempts in the 1970s to influence the disease used first ablation and then stimulation of various BG subdivisions [19]. Remarkable benefits were obtained occasionally by both strategies, but the deep location of relatively small targets and the primitive state of imaging (before CT and MR) led to inconsistent results. Stereotaxic ablations had the disadvantage of being fixed and irreversible, whereas electrical stimulation (Figure 1) could be turned on and off and tuned both intraoperatively and postoperatively to compensate for errors of placement and variations in the individual patient's anatomy and pathophysiology. Widespread clinical adoption of deep brain stimulation followed the development of sophisticated imaging combined with accurate and scaleable neuroanatomical atlases and electrophysiological methods to navigate intraoperatively by recording and stimulating BG neurons in awake patients performing simple motor tasks [20]. Empirical clinical success has motivated basic research into the unusual signal processing in the BG, which mostly employ inhibitory circuits to modulate spontaneous activity of intrinsic neurons. This complicates understanding of mechanism because stimulation in a given nucleus may activate incoming axons that inhibit its neurons rather than or in addition to exciting the cell bodies themselves.

Current research and development in DBS is concentrated on several remaining challenges and opportunities:

- Despite the improved targeting methods, electrodes are not always positioned to give optimal relief, so more sophisticated multicontact electrode arrays and multichannel stimulus generators may provide more opportunities to steer the stimulation currents postoperatively. This often entails making electrode contacts smaller and charge densities higher, so electrical safety must be reconsidered [21]
- For reasons that are not well understood, DBS usually requires continuous stimulation with fairly high charge pulses (~3–6 V ≈ 3–6 mA × 60–120 *μ*s) at high frequencies (>120 pps). This requires large implanted power cells that need frequently to be replaced (every 2–4 years) or recharged inductively. It may be possible to gate or titrate stimulation based on electrical or mechanical signals associated with the tremors that accompany PD in most patients [22]
- The BG connect with almost all cortical areas and have been implicated in various manifestations and pathologies of human intent besides the motor akinesia of PD, including obsessive-compulsive disorder, obesity, addictions, and depression. Finding targets for DBS to treat these disorders is complicated by the lack of immediate outcome measures that can be used to place electrodes and adjust stimulus parameters [23, 24]. It is difficult to exclude placebo effects from long-term studies of psychological disorders

### 2.3. Cochlear Implants

The basic function of the cochlea is spectral decomposition of soundwaves and transduction into tonotopically arranged neural action potentials from the auditory nerve to the brain. This was well known in the 1960s when clinicians first attempted to use electrical stimulation to restore hearing, despite the misgivings of prominent basic scientists in this field [25]. The first clinical device was a single channel stimulator that created an analog reconstruction of the acoustic waveform by modulating an inductively transmitted carrier, ignoring the science completely [26]. While it could not provide understandable speech, it did provide surprisingly useful cadence and loudness information that helped profoundly deaf patients attend to and recognize environmental sounds and enhance their speech reading (aka lip-reading) [27]. This motivated several international research groups to develop multichannel electrode arrays and stimulators that could take advantage of the tonotopic cochlea to provide multichannel spectral information (Figure 1).

Cochlear implant technology has now converged on several design principles that should have been apparent from biophysical theory rather than the expensive trial-and-error clinical experimentation that characterized the first two decades of this industry. In particular, it took a long time to abandon the delivery of analog waveforms to the electrodes and instead adopt pulsatile stimulation that provided explicit control of the all-or-none action potentials used to convey information throughout the nervous system [28]. It was well known that only a few channels of fairly broad spectral information would convey understandable speech [29], but early work on speech processors was often devoted to preprocessing the speech signals into high level percepts such as formants instead of simply delivering the raw spectral information [30]. The electronics technology was often focused on the addition of more channels rather than more selectivity of those channels. Because the sound frequencies relevant to speech are represented along a limited extent of the cochlea (~10–26 mm depth from the round window), increasing the number of electrodes means spacing them closer together, resulting in overlaps in neural excitation from adjacent channels. Studies of the speech perception as a function of number of channels stimulated have shown that intelligibility saturates at 6–8 channels [31], consistent with systematic electrophysiological and psychophysical studies of the spread of excitation from electrodes. Nevertheless, commercial cochlear implants now have 12–24 channels, greatly increasing their complexity and vulnerabilities. Early attempts to place electrodes in the auditory nerve itself were unsuccessful because the nerve fibers conveying widely different sound frequencies are packed even closer together in the nerve [32], a lesson that appears to have been forgotten in recent attempts to resurrect this strategy.

Cochlear implants are now a mature and successful technology and the treatment of choice for most patients with acquired (postlinguistic) or congenital (prelinguistic) sensorineural deafness. Performance in adults with acquired deafness remains surprisingly variable for reasons that remain unclear [33], but most achieve sufficient understanding of speech to function in the hearing world either immediately or after a few months of relearning. Performance in patients who lost hearing before language development is generally quite good but only if they are implanted at an early age (before age 4 and generally before age 2); this appears to reflect a critical period for normal development of the auditory nervous system. Even the best performers still have demonstrable hearing deficits in acoustically challenging environments such as background noise. The minority of deaf individuals who are not candidates for cochlear implants face a declining support system for sign language.

Current research and development in auditory prostheses is concentrated on several remaining challenges and opportunities:

- The auditory nervous system is designed to detect fine temporal structure in neural spike activity. Electrical stimulation produces very strong phase-locking responses [34] that are unrelated to the acoustic signal and its traveling wave on the basilar membrane, which may contribute to perceptual problems [33]. High frequency stimulation may mitigate some of these problems by reducing the inappropriate phase-locking [35] but at the cost of substantially higher power consumption that has not substantially improved speech perception [36]
- For various anatomical reasons, it is particularly difficult to obtain good spectral representation of the lowest frequencies of speech sounds (200–600 Hz), but these frequencies are often preserved in patients with acquired sensorineural deafness, which tends to spread from high to low frequencies. The original cochlear electrode arrays and surgical technique tended to damage any remaining hair cells, but modified designs have had some success in preserving this residual acoustic hearing so that the cochlear implant can be combined with a conventional hearing aid [37]
- The central neural pathways that process auditory information tend to combine spectral signals from both ears, at least in part to provide stereophonic localization of and attention to specific sound sources. Patients implanted with bilateral cochlear implants appear to obtain benefits, particularly in noisy environments, although insurance providers remain skeptical of the cost-benefit
- Patients with lesions of the auditory nerve itself cannot obtain benefit from cochlear implants. Despite the relative rarity of this bilaterally, substantial research has been devoted to electrical stimulation of the next stage in signal processing in the cochlear nucleus. Unfortunately, this structure is very difficult to access surgically, has a complex set of tonotopic representations in various subdivisions, and is often damaged or distorted by the benign neurofibromatous tumors that are the original cause of deafness in many such patients [38]

### 2.4. Artificial Eyes

The concept of using multichannel neural stimulation to convey visual information was one of the very first to capture the imagination of the pioneers of neural prosthetics in the 1960s, over the objections of basic scientists then working on vision [39]. Initial attempts were directed to arrays of electrodes on the pial surface of the primary visual cortex (V1 = Brodmann area 17). Stimulation of a single electrode required rather high charge delivery (1-2 mA × 0.1–0.2 ms) but produced surprisingly focused and retinotopically organized percepts called phosphenes [40, 41]. Neuroscience was just starting to understand the surround-inhibitory circuitry of cortical columns that allowed widespread activation of relatively deep pyramidal cells to be perceived as punctate phosphenes. Unfortunately, simultaneous or interleaved pulsatile stimulation of multiple electrodes resulted in unpredictable spatial and temporal interactions in that circuitry, defeating the notion of reconstructing an image from a pixel-like array of such electrodes and their phosphenes [42]. The high power levels required for cortical surface stimulation also raised questions of electrochemical and thermal safety. Arrays of penetrating microelectrodes were starting to be used to record and stimulate cortical neurons chronically, providing the means to excite smaller populations of neurons confined to individual cortical columns with much lower power [43]. A few experiments with acute and subacute microstimulation of visual cortex in awake humans confirmed that stable and combinable phosphenes could be obtained [44, 45], but extending the technology and surgical techniques to provide sufficient numbers of channels for useful vision remains daunting. In addition to mechanical challenges for leads, connectors, and packaging, the size of the microelectrode tips tends to decrease even faster than the required charge of the stimulating pulses, raising dangers of electrochemical damage if charge-density exceeds the limits of biocompatible electrode materials, which are now well understood [46, 47].

**Figure 2:**
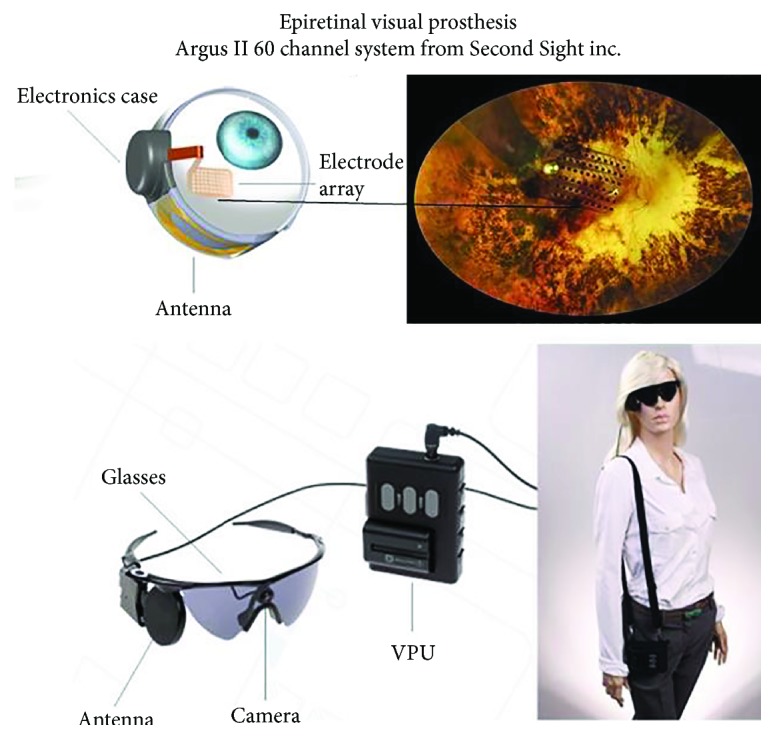
Epiretinal visual prosthesis consists of a highly flexible electrode array tacked onto the inner surface of the retina, which is connected to a multichannel stimulator that is strapped to the eyeball and receives power and command signals from an external video camera and video processing unit (VPU) via an externally generated radio-frequency magnetic field. Photos used with permission of the manufacturer, Second Sight Inc., Sylmar, CA 91342.

As progress on cortical visual prostheses dragged to a halt, interest arose in the eye itself as a more surgically accessible site for electrode arrays, at least in patients with selective degeneration of the photoreceptors but preservation of the retinal ganglion cells that make up the optic nerve [48]. Placing a few electrode contacts in a nerve cuff around the optic nerve produced the biophysically predictable result of very large phosphenes unsuitable to provide functional vision [49]. Placing an array of photocells inside the retina produced the biophysically predictable result of generating insufficient charge to activate the small unmyelinated cells of the retina; active amplification of photodiodes produced useful visual percepts, but the flexible active circuitry remains difficult to protect from corrosive body fluids [50]. Placing electrodes on the surface of the retina produced phosphenes at high but reasonable stimulus charge levels but only if they were in close contact with the delicate retina [51]. This gave rise to a substantial academic research and industrial development effort to provide a sufficient number of channels to provide useful vision. First 16- and now 60-channel systems implanted in patients (Figure 2) have produced some appreciation of high contrast, large, and/or looming objects but not appreciable visual acuity [52]. Detailed studies of the often large and elongated phosphenes evoked by each channel suggest that retinal ganglion cells are excited not just at cell bodies that are under a given electrode contact but also via their output axons that traverse the surface of the retina and under the electrode contact [53]. This agrees with a recent biophysical analysis of the excitability of these cells [54], although the applicability of that analysis is complicated by the extensive but highly variable degeneration of the retinal interneurons that are known to set the resting polarization level of the retinal ganglion cells. The phosphene problem might be mitigated by dense arrays of penetrating electrodes in much closer proximity to the ganglion cell bodies, but the fabrication and biocompatibility challenges are daunting. Subretinal arrays have been reported to generate more regular and combinable phosphenes [55] and perhaps more useful functional vision [56] than epiretinal arrays, but they pose larger engineering and surgical challenges to achieve high reliability.

It remains to be seen whether the clinically obtainable benefits from the available technology for electrical stimulation result in a commercially viable visual prosthesis. Recent development of optogenetic methods to incorporate photoreceptive channels into nonreceptor neurons such as retinal ganglion cells seems particularly promising in a system that normally transduces focused images into topically mapped neural activity. In principle, it could provide high spatial acuity without implanting complex and delicate electrode arrays [57]. Developing the requisite genetic engineering for humans and demonstrating its safety present large and unknown challenges.

### 2.5. Neuromuscular Stimulation

**Figure 3:**
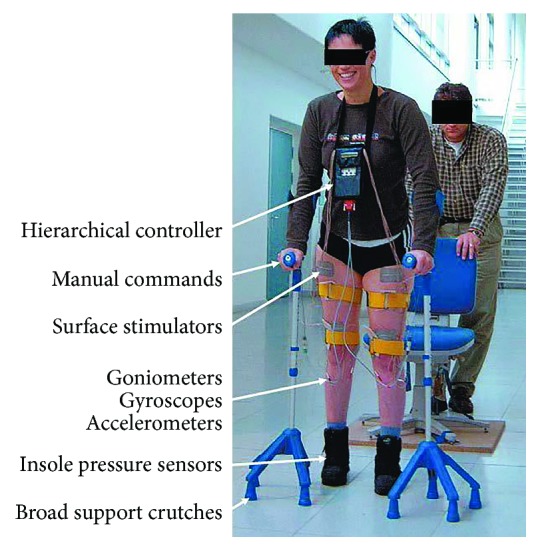
Functional electrical stimulation (FES) research system for paraplegic locomotion, adapted from [58].

**Figure 4:**
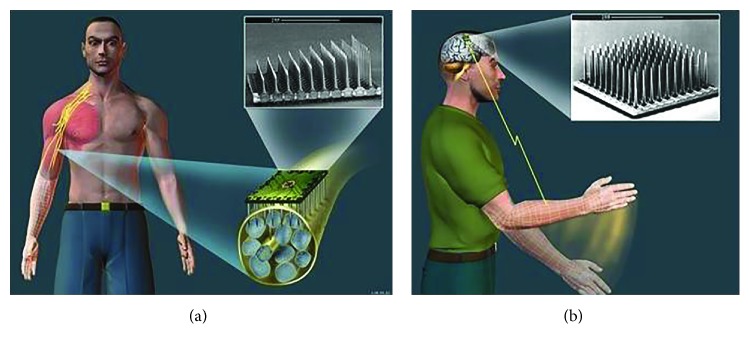
Penetrating microelectrode arrays to stimulate and/or record activity in individual neurons in peripheral nerves (a) [114] and cerebral cortex (b) [115]. Command signals from peripheral motor axons could be used to control prosthetic limbs, and stimulation of somatosensory afferents could provide perceptual feedback. Command signals from motor cortex can be used to control prosthetic limbs for amputees or neuromuscular stimulation for quadriplegic patients. Illustration provided courtesy of The Johns Hopkins University Applied Physics Laboratory.

Restoring muscle contraction in patients with paralysis has been an obvious clinical application for electrical stimulation since Luigi Galvani first discovered bioelectricity in frog muscles over 200 years ago [59, 60]. Early electrical technology gave rise to a wide range of quack-medicine instruments and treatments for everything from arthritis to stroke [61]. The early focus of neurophysiology on sensorimotor spinal function and control led to a particular emphasis on restoring walking in paraplegic patients with spinal cord injuries [58, 62] (Figure 3) and also reach and grasp function in quadriplegic patients [63]. Various academic and commercial schemes over the past 40 years have all foundered on a large number of fairly obvious problems:

- Functional use of the limbs requires selective and precise activation of a large number of widely distributed muscles. Muscle fibers themselves are relatively inexcitable [64], so stimulation must reach intact motor axons, which tend to lie deep to the skin and muscles and in mixed peripheral nerves that innervate multiple muscles. Transcutaneous electrodes on the surface of the skin do not achieve sufficient selectivity or reproducibility and produce massive stimulation of cutaneous afferents. Percutaneously implanted intramuscular wire electrodes pose unacceptable risks of breakage and infection [65]. Surgically implanted intramuscular or epimysial electrodes require massive surgery to route to all required muscles [66]. Surgically implanted cuff electrodes with multiple contacts on proximal nerve trunks may not produce sufficiently selective muscle activation and tend to recruit the largest and most fatiguable motor units at the lowest stimulus intensities [67]
- Useful movements require command signals from the patient that specify the details of the desired limb movement and an inverse model of the musculoskeletal plant and electrical recruitment scheme to compute the pattern of muscle stimulation required to achieve that movement [68]. Attempts have been underway for decades to record and decode command signals from the cerebral cortex using chronically implanted microelectrode arrays (see Brain-Machine Control and Figure 4), but the codes remain unclear and problems with biocompatibility cause signal degradation over months instead of the decades required for a clinical prosthesis
- Stable movements require continuous feedback control from proprioceptive and cutaneous afferents. Like the motor neurons to the muscles, these are mostly still present caudal to the spinal injury but chronically implantable means to record and decode tiny and highly intermixed sensory signals have yet to be reliable enough for clinical use [69]. Residual spinal circuits are a source of spasticity that interferes with all forms of rehabilitation [70]
- Locomotion presents special requirements and hazards. Balance depends on vestibular feedback, which is unavailable. Falling is likely to result in injury, with implicit product liability. Regaining upright posture after a fall is an even more complicated muscle coordination problem than regular gait. Useful locomotion requires some attention to energetic efficiency, which intact humans achieve by sophisticated dynamic transfers of momentum among continuously moving limb segments [71]. All of these requirements and hazards have already been addressed by a simple, inexpensive, and noninvasive prosthesis called the wheelchair

One successful application focuses on a very localized problem—foot drop in the weak leg of a stroke patient [72]. Only a couple of synergist muscles in the anterior shank need to be stimulated sufficiently but not precisely to dorsiflex the ankle and clear the ground during the swing phase of walking. The timing of this stimulation can be determined by sensing mechanical features of gait such as the pressure at the heel or the forward tilt of the shank. Both transcutaneous and implantable stimulators are commercially available.

Obstructive sleep apnea is another relatively localized functional problem that has been addressed by stimulating the hypoglossal nerve to the tongue. The technology has been complicated by the complex neuromuscular architecture of the tongue innervation [73] and by the perceived need to time the stimulation to coincide with the onset of obstructed inspiration [74, 75]; other approaches have been suggested [76].

Ironically, there are many clinically important musculoskeletal problems that arise from lack of muscle exercise rather than the lack of functional movement and that may be much more easily addressed with neural prostheses. Electrical stimulation can provide well-controlled, virtually effortless exercise whose benefits should be as effective as voluntary exercise without requiring the command and feedback signals needed for accurate functional movements [77]. Transcutaneous electrical nerve stimulators (TENS) must generally be applied by a trained therapist who can adjust electrode locations and stimulus parameters to activate the correct muscles while managing often unpleasant cutaneous sensations. If such therapeutic electrical stimulation (TES) can only be applied in a clinic, it is usually not cost-effective to provide sufficient exercise to be useful. Surgically implanted stimulators could be programmed for use by the patient at home, but these applications rarely justify the associated expense and morbidity.

**Figure 5:**
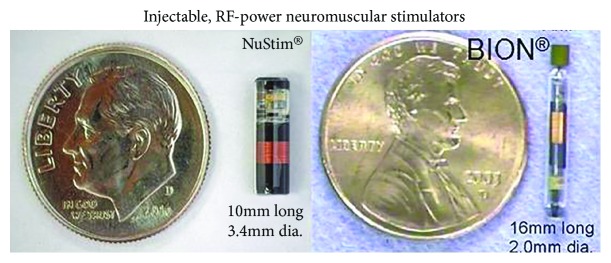
Two types of single-channel monolithic neuromuscular stimulators that can be implanted into individual muscles or near nerves, where they generate well-controlled stimulus pulses that are powered and commanded by an externally generated radio-frequency magnetic field.

Microstimulators have been developed that can be injected into muscles as an outpatient procedure and receive their power and command signals by telemetry from an external transmitter placed near the implants during daily exercise sessions at home [78] (Figure 5). Stroke patients with one paralyzed arm can still use their good arm for most activities, but they suffer from chronic shoulder pain and flexion contractures of the hand in the unused paralyzed arm. Spinal cord injured patients have a high incidence of pressure ulcers that arise from loss of mechanical padding as muscles atrophy from disuse [79]. Orthopedic patients with painful arthritis or requiring immobilization after a surgical repair experience loss of muscle strength that interferes with their rehabilitation [80]. Because microstimulators are novel Class III medical devices, regulatory and reimbursement approval has inhibited the development of commercial systems for these therapeutic applications.

### 2.6. Urinary Function

Urinary incontinence is a common clinical complaint. Similar symptoms may arise from very different underlying pathologies that tend to require rather different treatment.

- Stress incontinence arises because the external urethral sphincter cannot generate sufficient closing force to prevent urine from leaking when abdominal pressure increases (e.g., coughing and picking up heavy objects). It is particularly common in women who have had damage to the pelvic floor musculature during childbirth, and it gets worse with soft tissue atrophy during menopause. It has long been known that voluntary exercise to strengthen these muscles (Kegel exercises) can greatly improve the majority of patients, but it is difficult to train them to do the exercises correctly and regularly for several months [81]. Transcutaneous stimulation applied via intravaginal or intrarectal probes is effective but unpleasant [82]. An injectable microstimulator is under development for this application (NuStim in Figure 5) [83]
- Urge incontinence (overactive bladder) arises because the bladder starts to contract reflexively with even small volumes of urine, and the sphincter muscle soon fatigues from continuous use. It is particularly common in young women and is associated with bladder infections that leave scarred and hypersensitive bladder walls. It can be treated by taking advantage of an inhibitory reflex from the cutaneous receptors in the clitoris or penis that is designed to prevent urine leakage during sexual activity, so stimulation must be applied continuously except when micturition is desired [84]. A surgically implanted stimulator targets these sensory afferents in the spinal nerves as they exit the sacrum (Figure 1). Because of anatomical variability of the various sensory and motor nerves in this location, about half of such patients cannot obtain sufficient relief without undesirable side-effects [85]. A percutaneously implantable microstimulator with a small rechargeable lithium cell can target the same sensory nerve fibers in the pudendal nerve in the pelvis, but there were problems with implanting and maintaining them at the correct location, and the tiny lithium cell must be recharged via an external coil every few days [86]. Some success has been reported for stimulation of low-threshold mechanoreceptors in the leg using either implanted or transcutaneous stimulators [84], probably because of segmental inhibitory circuits similar to the gate theory for pain modulation
- Overflow incontinence arises because the patient cannot switch the urinary tract out of its normal state of maintaining continence by reflexive closure of the sphincter when a bladder contraction occurs; the bladder fills and pressure increases until urine leaks past the closed sphincter. It is particularly common after spinal cord injury and some neural degenerative diseases that interrupt the ability to sense bladder fullness and/or generate the descending command from the brain that inhibits the sphincter reflex. Various attempts have been made to stimulate in and around the spinal cord and sacral roots to find a site that would replace the missing signal but with inconsistent results [87–90]

### 2.7. Gastrointestinal Function

The stomach and intestines have phylogenetically old but very sophisticated neuromuscular systems to generate and coordinate the various motility and secretary functions required for efficient digestion of food and removal of waste products. The muscles are tonic rather than twitch type; they generate complex and often spontaneous states of stiffness and/or active contraction. They are coordinated by small neurons in parasympathetic ganglia within the walls of the gut and sympathetic ganglia deep in the abdomen. The control loops are modulated by many types of mechanical and chemical sensors in the gut, circulating hormone levels, and state changes tied to patterns of use (e.g., foods ingested and the microbiome living in the gut) and circadian rhythms. Many details of gastrointestinal control are just starting to be understood. Clinical measurements of function are difficult or unavailable or only indirectly related to physiological function [91].

The goal of electrical stimulation in the gastrointestinal system is usually to modulate the functional state of the neuromuscular system that remains in the patient, rather than to explicitly take control of the details of the physiological processes. Experiments to demonstrate the effects of systematically varied interventions such as electrical stimulation are difficult to conduct because the physiological functions normally operate once a meal or once a day. Furthermore, they may operate differently in different species and in what appear to be the same pathological states of different patients. As a result of these challenges, products to modulate gastrointestinal function have generally been based on highly limited experiments in animals followed by largely empirical development of the best methods of practice for the location of electrodes, the stimulus parameters, and outcome measures that seem to correlate with patient satisfaction [92–95].

Clinical disorders that have been treated with modulatory electrical stimulation:

- Gastroparesis is a failure of peristalsis to properly empty the stomach of partially digested food. It occurs commonly in patients with diabetic neuropathy of the parasympathetic vagus nerve that normally provides some neuromodulatory bias to the neuromuscular system in the stomach walls. This is particularly dangerous because these patients must estimate the rate of food digestion and resulting blood glucose levels to decide on their insulin dosage. Various sites and patterns of electrical stimulation have been used to convert activity patterns of the smooth muscles of the stomach from mixing action to waves of peristalsis
- Obesity has been treated with a variety of electrical stimulation targets and patterns to reduce appetite by inhibiting gastric emptying. Clinical results are similar to gastric banding [96]
- Lower gastrointestinal tract dysfunctions include both hypomotility (constipation) and hypermotility (diarrhea and dumping syndromes). Digital stimulation of perianal cutaneous mechanoreceptors has long been used by paraplegic patients to stimulate fecal emptying, providing a basis for transcutaneous electrical stimulation [97]. Reduced intestinal motility has been noted in animal experiments on gastric stimulation, but clinical syndromes are mostly transient and/or respond well to drugs

### 2.8. Electroceuticals for Autonomic Modulation

The gastrointestinal applications are one of the many physiological processes that are known to be controlled by the autonomic nervous system but for which the nature of the control is just starting to be understood. Pharmaceutical companies are getting into the field of “electroceuticals” in which electrons are treated like a neuromodulatory drug that can be delivered locally to targets identified through basic physiological research. Several clinical disorders have been targeted:

- Inappropriate or excessive immune reactions lead to a variety of autoimmune disorders such as rheumatoid arthritis and septic shock during otherwise manageable bacterial infections. Recently, it has been discovered that activity in parasympathetic efferent neurons in the branch of the left vagus nerve that innervates the spleen has a powerful inhibitory effect on the proliferation of the immune system cells responsible for some of these disorders [98]. Systematic neurophysiological studies in animals with endotoxemia [99] and patients with rheumatoid arthritis [100] identified long-lasting effects mediated by relatively large myelinated nerve fibers that are easily stimulated electrically. Because very little stimulation power is needed only intermittently, the entire device including a rechargeable lithium cell can be made small enough to sit with the electrodes inside a silicone nerve cuff that is implanted directly on the nerve in a minor surgical procedure (https://setpointmedical.com/technology/)
- The distribution of blood flow within and among organs of the body is controlled by smooth muscle sphincters on the arterioles whose contractile state is governed by both local and central chemical sensors and neural reflex loops. Clinical problems related to inadequate blood flow include cardiac angina, cerebral ischemia, and diabetic foot ulcers. The nerve fibers responsible for these functions tend to run with the blood vessels and are mostly small and often unmyelinated, making them difficult to excite electrically. Nevertheless, attempts to find useful and accessible targets, mostly in the spinal cord, are underway based largely on empirical strategies [101]. Electrical stimulation of the parasympathetic sphenopalatine ganglion can increase cerebral blood flow, motivating animal studies to mitigate ischemic stroke [102] and, paradoxically, clinical treatment of cluster headaches [103, 104]

### 2.9. Epilepsy Control

There are various forms of epilepsy based on the specific pathology of the neurons, the location of the pathology in the brain, and the tendency for epileptic activity to stay localized or spread like a wildfire. Most types of epilepsy in most patients are reasonably controlled by drugs or by surgical removal of an accessible focus of seizure activity. Some patients, however, have refractory, frequent, and severe seizures that interfere with normal life and risk mechanical damage to the body and hypoxic damage to the brain and heart when seizures cause uncontrolled movements or interfere with breathing. The frequency of such seizures may be highly variable over time and among patients. It is often influenced by poorly understood environmental and emotional factors, making it difficult to design well-controlled studies of the efficacy of treatment.

Early attempts to treat the most severe patients were directed toward stimulating the cerebellar cortex, whose output Purkinje cells generate inhibition of the neurons to which they project [105]. The hope was that this added inhibition would generally down-modulate the excitability of the brain and reduce the hyperexcitability responsible for the initiation and spread of seizures. There was considerable anecdotal evidence that it worked well in some patients but not at all in others. The positive results were confounded by a strong placebo effect in which patients subjected to highly invasive surgical implantations had large reductions in their seizures before the stimulation was even turned on [106]. The negative results were confounded by post mortem studies that revealed that many patients with severe grand mal seizures had widespread loss of the Purkinje cells as a result of repeated bouts of hypoxia. Despite many clinical reports of efficacy, the products were withdrawn [107].

Over the past 25 years, patients with intractable epilepsy have been treated with a surgically implanted stimulator on the left vagus nerve in the neck. The original research was based largely on a hunch (J. Zabara, U.S. Patent #4,702,254, 1987) because the known neuroanatomy and physiology of the vagus nerve contained nothing to suggest that it could influence the corticothalamic circuits responsible for epilepsy. The original hope of being able to use such stimulation to directly abort seizures did not pan out. Continuous stimulation of some unknown subset of vagus nerve fibers now appears gradually to decrease the frequency of epileptic seizures in at least some patients [108], a difficult claim to substantiate given the high intrinsic variability of the disease. Various deep brain and cortical structures have also been stimulated chronically to reduce epileptic seizures [109].

Responsive stimulation for epilepsy seeks to abort seizures before they progress. Implanted systems include both cortical recording electrodes (ECoG) to detect incipient seizures and stimulating electrodes at the seizure focus to deliver high frequency bursts [110, 111]. Seizures were originally thought to result from inadequate or fatigued GABAergic inhibitory neurons within the cortex, but susceptibility and pathophysiology may depend on genetic variants of ion channels [112, 113]. Electrical stimulation tends to excite the large pyramidal output cells, which have recurrent collaterals that excite these inhibitory interneurons, so it is at least theoretically possible that properly timed and located stimulation could abort the seizure if delivered soon enough. This would suggest that the clinical benefits should appear immediately, but the literature describes gradual improvements over years for both responsive and continuous stimulation in various sites, consistent with trophic effects on the target neurons but difficult to disentangle from symptomatic variability.

### 2.10. Brain-Machine Control

When active research on neural prosthetics started in the 1960s, there were visions of sophisticated bidirectional exchanges of information between any part of the nervous system and electronic and computational technology that would be able to work hand-in-hand with it. Half a century later, there is a more nuanced view of what is feasible and which clinical problems warrant the complexity, expense, and morbidity associated with such invasive treatment.

One rare but particularly desperate condition is “locked-in” syndrome, in which the cognitive ability of the brain is intact but the motor output pathways are completely blocked. Academic research on extracting information from EEG signals has resulted in commercial systems that can be used at home to steer a computer cursor by highly motivated patients and therapists willing to deal with complex donning and calibration procedures [116]. There have been several laboratory demonstrations in which brain signals recorded from implanted macro- or microelectrodes have been used to control robotic arms, but these are far from commercialization [68, 117–121]. Electrocorticogram signals from arrays on the pial surface require invasive implantation and communication methods and have yet to demonstrate substantially better information transfer than EEG. Penetrating arrays of microelectrodes can record discriminable unitary activity from individual neurons (Figure 4), which would be expected to provide more detailed information about intention and control, but recordings are unstable [122]. Tiny movements of the electrode tips cause abrupt loss or misidentification of previously recorded neurons, so control systems must be continuously retrained. Longer term accumulation of connective tissue around foreign bodies reduces the amplitude and discriminability of the neural signals over periods of weeks to months [123–125]. Dexterous manipulation of objects normally depends strongly on tactile feedback, which is just starting to be incorporated into prosthetic hands and telerobots as subconscious reflexes [126] or electrical stimulation of remaining sensory pathways in peripheral [127] or central nervous system [128, 129].

### 2.11. Mental Enhancement

More recently, there has been interest in achieving even tighter integration between higher-level brain functions such as memory and cognition and computers capable of performing similar tasks using adaptive neural networks. This might be used to restore lost function [130] or to enhance normal function. Even if the above-noted limitations in the interface technology could be overcome, perhaps by radically new technologies such as optogenetics [131, 132], clinical applications will depend on understanding the ways in which information is coded, communicated, and decoded in the targeted neural subsystems. Theories of these processes have been driven by recordings of single-unit activity during the performance of highly circumscribed tasks by chronically instrumented nonhuman primates. If the neural activity has some statistical correlation with the behavioral activity or external stimuli, it is said to “encode” that activity or stimulus. That does not mean, however, that stimulating such neural activity will actually regenerate the original behavior or percept because it represents only a tiny fraction of the distributed neural activity in the brain, and it may be monitoring rather than controlling the mental function.

At the opposite technological extreme, there has been much recent interest in transcranial direct current stimulation (tDCS), in which very low currents (typically 1-2 mA) are delivered across the entire cranium by large skin electrodes driven by a simple battery. This has become something of a do-it-yourself fad with anecdotal stories of nonmedical claims regarding enhanced mood and concentration. Scientific studies have been mostly small, poorly controlled, and often conflicting in outcomes, recently reviewed in [133]. The complex gross anatomy and widely differing conductivities of the various tissues in the path (skin, galea, skull, dura mater, cerebrospinal fluid, gray matter, and white matter) will result in current densities among neurons that are minuscule, diffuse, and unpredictable. The supposed mechanism of action is widespread subliminal polarization of dendritic trees, but any such effects are complicated by the highly variable orientation of neurons in the brain as a result of the deeply folded gyri of cerebral cortex. Galvanic vestibular stimulation (GVS) is a variant of tDCS that results in more obvious sensations of tilt and secondary postural responses. GVS has a more plausible mechanism of action in the selective flow of current through the conductive fluids of the semicircular canals and the exquisite sensitivity of hair cells, which respond similarly to hydraulic eddy currents induced by normal head motion and by caloric (thermal) stimulation of the ear canals [134]. Whether this is useful therapeutically remains to be determined.

tDCS is often lumped with repetitive transcranial magnetic stimulation (rTMS), a similarly noninvasive technology to modulate neural activity that has also been applied to a wide range of psychiatric and mood disorders including depression, autism, anxiety, and restless leg syndrome as well as other dystonias [135]. Such lumping is inappropriate; single-pulse TMS utilizes a well-defined technology and well-documented mechanism of action that has made it a valuable diagnostic and research tool for tracing corticospinal and other pathways [136]. Because rTMS requires expensive equipment operated by professionals, its use in treatment has been limited to relatively infrequent therapy sessions in small academic studies whose clinical significance is difficult to assess.

## 3. Discussion

### 3.1. Strategies for Research and Development

#### 3.1.1. Clinical Shots in the Dark

Many of the clinical successes described above started as crude schemes concocted by clinicians trying to solve clinical needs with little understanding of either the then-existing science or technology. Many academic scientists and the funding agencies that they control disdained such naïve efforts and became involved only when primitive interventions achieved unexpected albeit limited success. It seems likely that some promising applications of neural prosthetics remain abandoned simply because such simplistic approaches produced exactly the failures predicted.

Anyone who has been involved in projects that required clinicians, scientists, and engineers has experienced the challenges inherent in such collaborations. The project usually requires high-level skills and experience from all three disciplines. Combining those backgrounds in one individual has become more rare as each has become more demanding. Suitable individuals from each discipline usually have deeply ingrained motivations, attitudes, languages, working styles, schedules, and reward systems that are unique to their disciplines. This may explain why the haphazard strategy of shots in the dark has not disappeared entirely.

A less generous interpretation is the recurring hope that electricity will magically cure complex disorders without requiring any understanding of the ever-more-sophisticated science and technology. Popular low-tech fads such as tDCS seem to be replaying the 1970s crazes for acupuncture and biofeedback therapy, both of which are also making comebacks [137]. Purveyors of complementary and alternative medicine make claims of cumulative treatment effects based on subjective outcome measures that are impossible to refute. It is paradoxical but true that rapid advances in science and technology tend to spark a backlash of those who reject objective data and instead demand the right to purchase treatments that are not approved or reimbursed through conventional channels. This is particularly likely to be the case for functional disorders related to lifestyle choices, for which modern medicine often has little to offer.

#### 3.1.2. Technological Solutions in Search of Problems

Virtually all of the systematic attempts and evolutionary successes described above stemmed from the many advances in electrodes and instrumentation developed over the past century as research tools for neurophysiologists: chronically implantable electrode materials, nerve-cuff electrodes, microelectrode arrays, low-noise amplifiers, biphasic stimulators, multichannel telemetry, etc. Basic scientists using those tools to investigate normal behavior would often be aware of clinical pathology in those systems, so it was natural to try to use the technology at hand to address clinical needs. Such attempts depend also on the state of knowledge about normal and pathological function, which might or might not be sufficient to achieve success even if the technology happens to be adequate. Most of the clinical successes described above arose from the fortuitous combination of primitive technology applied to neural systems that were amenable to simplistic interventions—essentially “low-hanging fruit.” Not only has that fruit been picked, but the rules governing the orchard have changed. The preclinical and regulatory effort now required to adapt, test, and validate research instrumentation even for feasibility studies in human subjects largely precludes such informal activities.

#### 3.1.3. Defined Physiological and Anatomical Targets and Bespoke Technology

It should now be possible and may even be necessary for new clinical neural prostheses to start with a careful analysis of a clinical problem from all the relevant perspectives identified below. This would ensure that available and important information is not overlooked and that the required research and development steps are prioritized so as to reduce systematically the anticipatable risks of failure:

1. The complete needs of patients and their caregivers faced with a clinical disorder in the context of their disabilities, lifestyles, disease progression, and aspirations
2. The incidence, prevalence, and current and prospective alternative treatments for the clinical disorder
3. The normal and pathological physiology relevant to the disability and its likely response to putative interventions
4. The anatomical and surgical accessibility of sites where neural prosthetic interfaces might produce a substantial net benefit with minimal side-effects and health risks
5. The currently available technologies for such interfaces
6. Missing pieces of information that might identify more suitable targets or enable simpler technology
7. Enhancements to currently available technology that might enable better modes of treatment with higher probability of better clinical outcomes or lower total costs of treatment
8. Feasibility and cost/benefit analysis for research to provide missing science and technology before starting product development

### 3.2. Key Tactical Issues for Successful Products

#### 3.2.1. Start Design Control Early

If a neural prosthesis project starts with an organized strategy to build a commercializable system to address a specific clinical problem, then the project can and should make use of certain tools that are now mandated by the medical device regulations of the US FDA and, increasingly, other regulatory agencies (capitalized items below). Chief among them is Design Control, a systematic methodology to pursue and document the steps listed above. Such documentation is now a required component of the application to market Class II and III medical devices, which include virtually all neural prostheses. A complete, documented, and approved Design Input Document then acts as a performance standard to which the final product can be tested. These verification and validation results become part of a Design Output Document, which includes the Device Master File, essentially the complete recipe for building and testing the manufactured product [138].

Neural prosthesis projects often start as unanticipated offshoots of basic research in academic institutions, as noted above. These organizations generally do not have Quality Systems or personnel familiar with the formal methodology of Design Control. The US FDA generally recognizes such feasibility research as part of a Concept Phase that is exempt from Design Controls even in commercial organizations. Nevertheless, the steps enumerated above come naturally (or should) to academic researchers used to scholarly examination of the scientific literature, logical experimental design, well-planned research proposals, and well-documented publication of results. Familiarity with Design Control helps to avoid tunnel vision about science and technology that may overlook critical aspects of patient needs, competing technologies, and clinical dissemination of new medical devices. Organizing the complete analysis in a form compatible with Design Control methodology will greatly facilitate acceptance by and progress of the commercial entity that must eventually turn the concept into a business.

#### 3.2.2. Anticipate Clinical Trials for Regulation and Reimbursement

Novel invasive medical devices (Class III in the US and European Community) generally require demonstrations of both safety and efficacy that, in turn, require clinical trials. Such clinical trials are often the most expensive, lengthy, and risky aspects of medical product development. Because separate government agencies handle approval to market (FDA in the US, notified bodies in Europe) and approval for insurance coverage (CMS in the US, National Health Services in Europe), the clinical data collection and submission processes have often been pursued separately and inefficiently. A successful medical product generally requires a careful analysis of the complete business model, which will influence both the design of the product and the claims that need to be supported by clinical data. Innovative medical devices often start with science and technology but commercial products need to consider much more [139].

One recurring theme in the history of neural prosthetics is the disappearance of modes of treatment and specific products that fall out of favor clinically without having definitive negative reports in the literature. The clinical applications for these technologies often involved desperate patients suffering from life-changing symptoms or disabilities. Both the patients and their caregivers were anxious to find mitigating treatments, companies needed to sell products, and journals wanted to publish highly cited papers. The pharmaceutical industry is already wrestling with the problem of unreproducible results in basic science and preclinical studies related to disease [140] and clinical trials of new drugs [141]. Treatments that do not actually live up to their claimed benefits will eventually fall out of favor through the cumulative effects of informal clinical experience, but the process is extremely inefficient for society and results in a concomitant loss of credibility that inevitably affects the acceptance of subsequent novel treatments.

In the examples above, three factors stand out as obstacles to definitive clinical trials:

1. Claims supported by subjective patient-reported benefits rather than objective outcome measures (e.g., analgesia and mood-elevation). These are obviously prone to placebo effects, which may be impractical or impossible to control with sham procedures when active devices are involved
2. Claims related to long-term use rather than immediate effects (e.g., chronic pain, epilepsy, and depression). Most chronic clinical problems tend to vary in their severity, and patients tend to seek treatment when they are at their worst and likely to improve spontaneously
3. Similar disease phenotype with various underlying pathophysiologies that cannot be identified clearly (e.g., epilepsy, stroke, and tinnitus). A treatment that is actually highly effective for one underlying cause will not be able to demonstrate statistical significance if there are many negative results in patients with other underlying causes

#### 3.2.3. Build Sustainable Interdisciplinary Teams

As noted above, clinicians, scientists, and engineers tend to have widely differing motivations, skills, and technical languages. Adding business and regulatory experts further complicates the functionality of the team. That probably explains why most neural prosthetic products have been commercialized by larger and older companies that already have both team leaders and team members who are comfortable in such environments. Unfortunately, such companies have large vested interests in technologies that they have developed and understand (e.g., pacemaker-like devices in Figure 1), which makes them resistant to innovative and even disruptive technologies that pose unknown risks to both their customers and their business models. Thus, it often falls to academic research groups and start-up companies to pioneer novel medical devices. Such teams tend to have relatively narrow expertise, at least initially. Fortunately, both universities and venture capital groups can usually draw on relatively large networks of people with wide ranging expertise. Making use of such resources often entails a dilution of both strategic and financial control, but it is more likely to lead founders to a smaller share of success than to full ownership of failure.

## 4. Prognosis for the Industry

The rapid increase in clinical trials of novel applications of neural stimulation will continue, but the percentage of successful commercialization seems likely to decrease. Part of that is because the low-hanging fruit has been picked, but it is also because reimbursers are becoming more demanding of cost-benefit data used to make decisions about coverage and payment. For chronic diseases and disabilities with high continuing care costs, even expensive treatments tend to be cost-effective but only if the treatment substantially reduces those costs and the decision-makers actually take them into account [142]. As the fundamental limitations of electrical interfaces become more apparent, academic attention will shift to optogenetic interfaces, but their uncertain safety and huge regulatory obstacles will delay such products for many years.
